# Antidepressant treatment as a modulator of inflammation in depression and heart failure

**DOI:** 10.1192/j.eurpsy.2026.10358

**Published:** 2026-07-31

**Authors:** A. K. Sikora, EPA Section on Consultation-Liaison Psychiatry and Psychosomatics

**Affiliations:** Ivano-Frankivsk National Medical University, Ivano-Frankivsk, Ukraine

## Abstract

**Abstract:**

Depression is common in patients with chronic heart failure (CHF) and is associated with poorer functional outcomes, reduced quality of life, and increased morbidity. Growing evidence indicates that inflammatory pathways may represent a shared mechanism linking depressive symptoms and cardiovascular disease progression. This presentation summarises clinical and translational findings on the role of antidepressant treatment as a potential modulator of inflammation in patients with comorbid depression and CHF. Focusing on multimodal antidepressants, I will discuss observed changes in biomarkers relevant to psychocardiology—including soluble ST2 and fibronectin—and their relationship with symptom severity and clinical status. The talk will address practical implications for consultation-liaison psychiatry, including psychopharmacological decision-making in cardiac patients, the potential value of biomarker-informed monitoring, and interdisciplinary collaboration with cardiology. By connecting inflammatory mechanisms with clinical outcomes, the presentation supports a more integrated, mechanism-informed approach to treating depression in CHF, aiming to improve both emotional well-being and cardiovascular care.

**Disclosure of Interest:**

None Declared

